# Rezatapopt and the Return of Targetable p53

**DOI:** 10.1002/mco2.70842

**Published:** 2026-06-19

**Authors:** Yuanze Luo, Assam El‐Osta

**Affiliations:** ^1^ Baker Heart and Diabetes Institute Epigenetics in Human Health and Disease Program Melbourne Victoria Australia; ^2^ Baker Department of Cardiometabolic Health The University of Melbourne Parkville Victoria Australia; ^3^ Department of Medicine and Therapeutics The Chinese University of Hong Kong Hong Kong SAR China

1

In a Phase 1 study published in *The New England Journal of Medicine*, Dumbrava and colleagues report objective responses with rezatapopt (PC14586), an oral small‐molecule p53 reactivator, in patients with TP53 Y220C–mutated solid tumors [1]. The study provides early clinical evidence that allele‐selective pharmacological stabilization of mutant p53 can generate antitumor activity and offers circulating tumor DNA kinetics as a potential pharmacodynamic readout for response and emerging resistance.

Rezatapopt represents a mutation‐selective p53 reactivation strategy rather than a broadly applicable p53‐targeting approach. The TP53 Y220C substitution creates a ligandable cavity within the p53 core domain, enabling rezatapopt to stabilize the mutant protein toward a more functional conformation. These findings support an emerging precision‐oncology framework in which selected tumor‐suppressor alterations may be therapeutically addressed through allele‐selective chemistry, while recognizing that this principle may not extend uniformly across TP53 mutations or tumor contexts.

p53 became famous not because it is complex, but because it is central. Lane's “guardian of the genome” framing captured the practical truth: p53 sits at the intersection of damage sensing, cell‐cycle arrest, and apoptosis, largely through transcriptional programs [2]. Levine and colleagues, even earlier, established p53 as a tumor suppressor whose inactivation by mutation is among the most common genetic events in cancer [3]. Those two ideas, centrality and ubiquity, made p53 biologically irresistible and therapeutically frustrating. The broader p53 network context—including stress sensing, transcriptional outputs, and feedback control—has been extensively reviewed and helps frame why “restoration” can be context‐gated [4].

The frustration was structural and pharmacologic. Tumor suppressors are loss‐of‐function lesions, and most p53 mutants do not present a stable pocket that small molecules can bind with both potency and selectivity. Y220C is different. It destabilizes the protein and creates a ligandable cavity. Rezatapopt exploits that geometry. The key conceptual upgrade is not “p53 is druggable.” It is: some p53 alleles create druggable geometry, and those alleles can be developed like precision targets—enroll by genotype, dose by exposure, and interpret outcomes through pathway execution, not histology. Phase 1 data are inherently limited, but this study yields operational signals that can be standardized.

First, dosing and administration behave like part of the mechanism: gastrointestinal tolerability (notably nausea and vomiting) and a food effect shape exposure, and the recommended Phase 2 regimen (2000 mg once daily with food) makes schedule and supportive care part of development rather than logistics.

Second, the study reported exploratory response enrichment among KRAS wild‐type tumors at active dose levels. This observation should not be interpreted as establishing KRAS status as a validated predictive biomarker, but it does support further investigation of pathway context. The capacity of restored p53 to induce cell‐cycle arrest or apoptosis is likely to depend on lineage‐specific transcriptional programs, co‐occurring genomic alterations, MAPK pathway activity, and survival bypass mechanisms. Accordingly, future studies should evaluate which molecular states permit pharmacological p53 restoration to translate into tumor control.

Third, the translational development of rezatapopt would benefit from pathway‐anchored pharmacodynamic readouts capable of distinguishing inadequate target engagement, incomplete transcriptional restoration, and restoration with downstream pathway blockade. Because p53 functions primarily as a transcription factor, pharmacodynamic assessment should include induction of canonical p53 target genes, such as CDKN1A and MDM2, together with apoptosis‐ or cell‐cycle‐associated markers that indicate biological execution rather than transcriptional induction alone. Where tumor biopsies are feasible, these analyses could be extended using single‐cell RNA sequencing to resolve treatment‐induced transcriptional states and tumor‐cell heterogeneity, and p53 ChIP‐seq to determine whether rezatapopt restores canonical or non‐canonical p53 occupancy at genomic regulatory elements. Where biopsies are not feasible, ctDNA kinetics may provide an accessible complementary readout of tumor response and emerging resistance. This mechanistic and translational framework is summarized in Figure 1.

**FIGURE 1:**
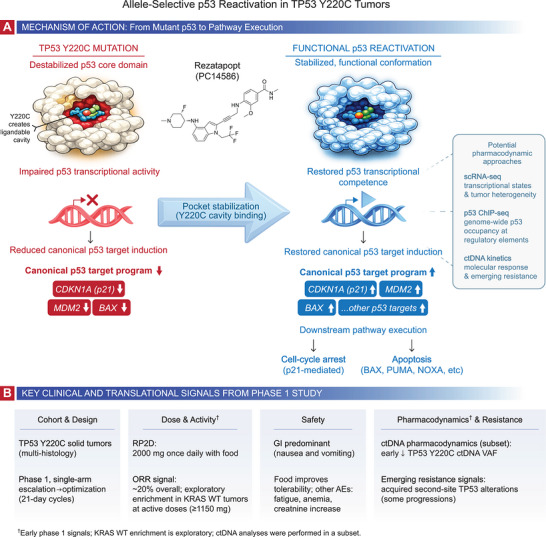
Allele‐selective p53 reactivation with rezatapopt (PC14586) in TP53 Y220C–mutant solid tumors. (A) Mechanistic model of rezatapopt‐mediated p53 functional reactivation. The TP53 Y220C substitution destabilizes the p53 DNA‐binding core domain and creates a ligandable cavity; this destabilization can impair p53 DNA‐binding and transcriptional activity. Rezatapopt (PC14586) is designed to bind the Y220C‐associated pocket and stabilize mutant p53 toward a more functional conformation. This may restore p53 transcriptional competence, including induction of canonical p53 target genes and downstream pathway execution through cell‐cycle arrest and/or apoptosis. Potential pharmacodynamic approaches to interrogate this mechanism include single‐cell RNA sequencing to resolve transcriptional states and tumor heterogeneity, p53 ChIP‐seq to assess genome‐wide p53 occupancy, and circulating tumor DNA kinetics to monitor molecular response and emerging resistance. (B) Key clinical and translational signals from the Phase 1 study of rezatapopt in patients with advanced TP53 Y220C–mutant solid tumors. The study used a single‐arm dose‐escalation and dose‐optimization design across multiple histologies, with a recommended Phase 2 dose of 2000 mg once daily with food. Objective responses were observed as an early efficacy signal, with exploratory enrichment among KRAS wild‐type tumors at active dose levels. Adverse events were predominantly gastrointestinal, including nausea and vomiting, with tolerability improved by administration with food. Circulating tumor DNA analyses in a subset showed early reductions in TP53 Y220C variant allele fraction, while acquired second‐site TP53 alterations in some progressing tumors suggest emerging resistance mechanisms requiring further evaluation. Clinical activity, KRAS wild‐type enrichment, and ctDNA pharmacodynamic findings should be interpreted as exploratory Phase 1 signals.

Because restored p53 activity may be insufficient to drive tumor regression in all cellular contexts, rational combinations should aim to increase DNA damage signaling, enhance apoptotic priming, or suppress survival pathways that blunt p53 execution. Such combinations should be prioritized when they strengthen measurable p53 pathway output rather than simply adding cytotoxic pressure.

Three combination classes are defensible, provided they are paired with falsifiable guardrails. Stress‐amplification partners could include selected DNA‐damaging or replication‐stress therapies, such as platinum agents, topoisomerase inhibitors, or radiotherapy, provided they increase tumor p53 pathway execution rather than systemic toxicity alone. Execution‐sensitization partners could include agents that increase apoptotic priming in p53‐restored cells. Bypass‐state partners, including MAPK pathway‐directed approaches in appropriate KRAS/MAPK‐active contexts, should demonstrate both pathway‐state modulation and downstream p53 execution in the same model. Classic MDM2‐p53 regulation illustrates a recurring problem in pathway pharmacology: upstream engagement can be measurable while downstream execution remains limited [4, 5].

The study establishes that allele‐selective pharmacological reactivation of TP53 Y220C‐mutant p53 can produce objective responses in patients selected by tumor genotype. However, it does not establish that p53 is broadly druggable across TP53‐mutant cancers, because Y220C is distinctive in creating a ligandable cavity. Nor does it define the resistance landscape at scale or demonstrate that pharmacological p53 restoration will produce equivalent biological consequences across tumor lineages.

The modest objective response rate despite genotype‐selected enrolment likely reflects the fact that TP53 Y220C is necessary for target engagement but not sufficient for uniform tumor control. Variable drug exposure, lineage‐specific transcriptional competence, co‐occurring oncogenic alterations, impaired apoptotic machinery, tumor heterogeneity, and emergent resistance may all limit conversion of p53 stabilization into durable regression. These limitations define a clear experimental agenda: standardizing pharmacodynamic readouts, identifying pathway‐state features that modify response, characterizing resistance mechanisms longitudinally, and designing rational combinations. The broader implication is that small‐molecule restoration of a tumor suppressor should be viewed not as a generic therapeutic class, but as an allele‐defined precision strategy whose success depends on demonstrating target engagement, transcriptional restoration, downstream pathway execution, and durable tumor control. Rezatapopt should therefore be developed as a pathway‐restoration therapy, with combinations advanced only when mechanistic linkage between engagement and execution is demonstrated.

## Author Contributions

Y.L. and A.E.O. conceived and wrote the manuscript, revised the manuscript, and approved the final version. All authors have read and approved the final manuscript.

## Funding

National Health and Medical Research Council (NHMRC) Clinical Trials and Cohort Studies Grant; APP2014763; NHMRC Fellowship (APP1154650; Breakthrough T1D (formerly JDRF) 3‐SRA‐2024‐1593‐A‐N.

## Ethics Approval

The authors have nothing to report.

## Conflicts of Interest

The authors declare no conflicts of interest.

## Data Availability

The authors have nothing to report.
